# Comparison of efficacy of nadroparin and fondaparinux sodium for prevention of deep vein thromboembolism in lower extremities after total hip arthroplasty and total knee arthroplasty: a retrospective study of 592 patients

**DOI:** 10.1186/s12893-024-02440-0

**Published:** 2024-05-18

**Authors:** Xiang Gao, Xiaowei Jin, Rui Huang, Zhenxing Li, Hanle Zhang, Pei Fan

**Affiliations:** 1 Department of Orthopedics, Yuying Children’s Hospital, The Second Affiliated Hospital of Wenzhou Medical University, No.109, Xueyuan West Road, Wenzhou, Zhejiang China; 2 Department of Rehabilitation, Yuying Children’s Hospital, The Second Affiliated Hospital of Wenzhou Medical University, Wenzhou, Zhejiang Province China

**Keywords:** Deep vein Thromboembolism, Nadroparin, Fondaparinux Sodium, Total hip arthroplasty, Total knee arthroplasty

## Abstract

**Objectives:**

To compare the efficacy of nadroparin and fondaparinux sodium for prevention of deep vein thromboembolism (DVT) in lower extremities after total hip arthroplasty (THA) and total knee arthroplasty (TKA).

**Methods:**

A total of 592 patients were enrolled in the study. Clinical data of patients who underwent total hip arthroplasty (THA) and total knee arthroplasty (TKA) in our hospital from December 2021 to September 2022 were retrospectively collected, which mainly included patients’ general information, surgery-related information, and DVT-related information. The patients were categorized into the nadroparin group(*n* = 278) and the fondaparinux sodium group(*n* = 314) according to the types of anticoagulants used. Anticoagulant therapy began 12–24 h after operation and continued until discharge. DVT prevalence between two groups was compared. The Statistical Package for Social Sciences (SPSS) software version 25 (SPSS, Armonk, NY, USA) was used for statistical analysis.

**Results:**

The prevalence of DVT in the nadroparin group and the fondaparinux sodium group was 8.3% (23/278) and 15.0% (47/314), respectively(*p* = 0.012). Statistical analysis showed that nadroparin group showed a lower prevalence of thrombosis than fondaparinux group (OR = 1.952, *P* = 0.012). Subgroup analyses showed that nadroparin group had a lower prevalence of DVT than fondaparinux group in some special patients groups such as female patients (OR = 2.258, *P* = 0.007), patients who are 65–79 years old (OR = 2.796, *P* = 0.004), patients with hypertension (OR = 2.237, *P* = 0.042), patients who underwent TKA (OR = 2.091, *P* = 0.011), and patients who underwent combined spinal-epidural anesthesia (OR = 2.490, *P* = 0.003) (*P* < 0.05).

**Conclusion:**

Nadroparin may have an advantage over fondaparinux sodium in preventing DVT in lower extremities after THA and TKA.

## Introduction

Venous thromboembolism (VTE), including deep venous thrombosis (DVT) and pulmonary embolism (PE), is a serious complication with high prevalence during hospitalization and perioperative period, affecting nearly 10 million people worldwide each year [1–3]. VTE is a multifactorial disease caused by the interaction of multiple predisposing factors, which can be transient or persistent, and strong transient predisposing risk factors such as major surgery, prolonged immobilization, and major trauma, accounting for approximately 20% of all VTE events [4]. In the absence of thromboprophylaxis, the prevalence of DVT after orthopedic surgery varies from 42 to 57% after THA and from 41 to 85% after TKA [5], and is as high as 60% after lower extremities orthopedic surgery within 2 weeks, of which 14% develop symptoms [6]. Compared with non-surgical patients, THA or TKA patients had the highest risk of VTE [odds ratio (OR) > 10] [7]. In addition, patients undergoing THA or TKA have a higher risk of VTE due to concurrent risk factors, such as age > 60 years, obesity [body mass index (BMI) > 30], diabetes, dyslipidemia and inflammatory diseases, and all the above are common risk factors for VTE and osteoarthritis [8].

Although VTE is a serious complication during hospitalization and perioperative period, it is preventable [6]. Effective anticoagulation prophylaxis for patients undergoing major orthopedic surgery can not only reduce the prevalence and mortality of VTE, but also alleviate pain for patients and reduce medical expenses [5, 9]. Current preventive measures include primary prevention, physical prevention, and pharmacological prevention, and pharmacological prevention is considered one of the most effective ways to reduce the risk of DVT in lower extremities [10]. Commonly used anticoagulants in hospital include unfractionated heparin (UFH), low-molecular-weight heparins (LMWHs), fondaparinux sodium and danaparoid [11]. Among them, LMWHs mainly include enoxaparin (low-molecular-weight heparin sodium) and nadroparin (low-molecular-weight heparin calcium) [11]. Nadroparin and fondaparinux sodium are two commonly used anticoagulants in clinical practice. However, the efficacy between nadroparin and fondaparinux sodium for prevention of DVT in lower extremities is still unclear.

Previous studies have found fondaparinux sodium to be superior to enoxaparin in preventing VTE [12, 13]. Related studies in China have shown that fondaparinux sodium is superior to LMWHs in preventing DVT in patients undergoing major orthopedic surgery [14], but this study does not limit the types of LMWHs. Some investigators have studied the prevention of thrombosis after minimally invasive esophagectomy(MIE) with nadroparin and fondaparinux sodium, and found that the prevalence rate of fondaparinux sodium was lower than that of nadroparin, so the investigators believe that fondaparinux sodium had similar efficacy in the prevention of thrombosis after MIE compared with nadroparin [15], but the sample size was small (*N* = 116). Therefore, we were curious about the performance of nadroparin and fondaparinux sodium in preventing DVT in lower extremities after joint arthroplasty.

The primary aim of this study was to retrospectively study the data of patients undergoing THA and TKA in the Second Affiliated Hospital of Wenzhou Medical University, in order to compare the prevalence of VTE with nadroparin and fondaparinux sodium for the prevention of VTE after THA and TKA. And the secondary aim was to explore the risk factors of VTE in each group.

## Materials and methods

### Study population

This study retrospectively analyzed 572 patients who underwent THA and TKA in the Department of Orthopedics of the Second Affiliated Hospital of Wenzhou Medical University from December 2021 to August 2022.

### Inclusion and exclusion criteria

The inclusion criteria in our study were: (i) patients who were admitted to our hospital from December 2021 to August 2022; (ii) patients who underwent THA or TKA during hospitalization; (iii) ultrasonography of lower extremity veins was performed before and after operation; (iv) no deep venous thrombosis occurred before operation; (v) nadroparin and fondaparinux sodium was used for postoperative prophylaxis; (vi) patients agree to receive anticoagulant therapy to prevent thrombosis and submit signed informed consent.

The exclusion criteria were as follows: (i) anticoagulants other than nadroparin and fondaparinux sodium were used; (ii) patients refused to accept anticoagulant therapy; (iii) patients with contraindications for anticoagulation, such as patients with a history of allergy to anticoagulants, acute bacterial endocarditis, thrombocytopenia, fresh bleeding, stroke within 3 months.

We reviewed a total of 676 patients who underwent THA and TKA in the Department of Orthopedics of the Second Affiliated Hospital of Wenzhou Medical University from December 2021 to August 2022. According to the inclusion criteria and exclusion criteria, 104 patients were excluded, and a total of 572 patients were finally included in this study.

### Data collection

#### Administration of anticoagulants

All admitted patients routinely assessed thromboembolic risk using a scoring scale. We also assessed patients for contraindications to anticoagulation. For patients with no contraindication to anticoagulation, Nadroparin (Fraxiparine, GlaxoSmithKline, UK) or Fondaparinux Sodium (Hengrui, Jiangsu, China) was administered subcutaneously on the first postoperative day to prevent DVT. Anticoagulant therapy began 12–24 h after operation and continued until discharge. In details, the nadroparin was applied as follows: 0.4 mL (4100AxaIU) per day, subcutaneous injection for 14 days beginning 24 h after discontinuation of anesthesia; The fondaparinux sodium was applied as follows: 0.25 mg per day, subcutaneous injection for 14 days beginning 24 h after discontinuation of anesthesia.

#### Diagnosis of DVT

DVT was diagnosed according to the criteria of Fraser et al. [16]. Doppler ultrasound was used to diagnose DVT. The patients were examined on the second day preoperatively and postoperatively.

#### General characteristics

We collected general information (gender, age, body mass index, etc.), current medical history, past medical history (diabetes, hypertension, coronary heart disease), postoperative Caprini score, drug precautions, surgery-related information (surgical methods, anesthesia methods, etc.), thrombosis prevalence (whether thrombosis occurred). The outcome variable was the occurrence of nosocomial thrombosis.

### Statistical analysis

The Statistical Package for Social Sciences (SPSS) software version 25 (SPSS, Armonk, NY, USA) was used for statistical analysis. The Shapiro-Wilks test was used to assess the distribution of the data. Continuous variables were expressed as mean ± standard deviation. Student’s t-test was used for parametric analysis of continuous variables and Mann-Whitney U test was used for nonparametric analysis of continuous variables. Chi-square test or Fisher’s exact test was used for categorical variables analysis. *P* < 0.05 was considered significant.

## Results

### Patients demography

After applying the inclusion and exclusion criteria, a total of 592 THA or TKA patients admitted to our hospital between December 1, 2021 and August 31, 2022 were included. 278 patients received nadroparin and 314 patients received fondaparinux sodium. The demographic data and baseline levels were recorded. The mean age was 68.85 ± 10.007 years in nadroparin group while 67.06 ± 10.688 years in fondaparinux sodium group. The proportion of males was 37.1% in nadroparin group while 29.9% in fondaparinux sodium group. In addition, we divided all patients into 3 age groups to balance baseline data for age. The detailed information about other clinical characteristics, including comorbidity, type of surgery and type of anesthesia between the two groups is exhibited in Table 1. Statistical analysis showed that baseline data were balanced and comparable between the two groups.

**Table 1:** Patients demography

	Nadroparin (*n* = 278) no. (%)	Fondaparinux Sodium (*n* = 314) no. (%)	*P*-value
DVT	23(8.3)	47(15.0)	0.012
Gender			0.067
Female-no.(%)	175(62.9)	220(70.1)	
Male-no.(%)	103(37.1)	94(29.9)	
Age (mean ± SD)	68.85 ± 10.007	67.06 ± 10.688	0.036
Age stratification			
≥ 80 year.-no. (%)	35(12.6)	25(8.0)	0.573
65–79 year.-no. (%)	160(57.6)	187(59.6)	0.325
<65 year.-no. (%)	83(29.8)	102(32.4)	0.155
Comorbidity			
Hypertension - no. (%)	131(47.1)	141(44.9)	0.589
Type 2 diabetes - no. (%)	45(16.2)	45(14.3)	0.530
Coronary heart disease - no. (%)	9(3.2)	2(0.6)	0.019
Type of surgery			0.051
THA- no. (%)	108(38.8)	98(31.2)	
TKA- no. (%)	170(61.2)	216(68.8)	
Type of anesthesia			0.243
General anesthesia- no. (%)	96(34.5)	123(39.2)	
Combined spinal-epidural anesthesia- no. (%)	182(65.5)	191(60.8)	

### DVT prevalence

**Fig. 1:**
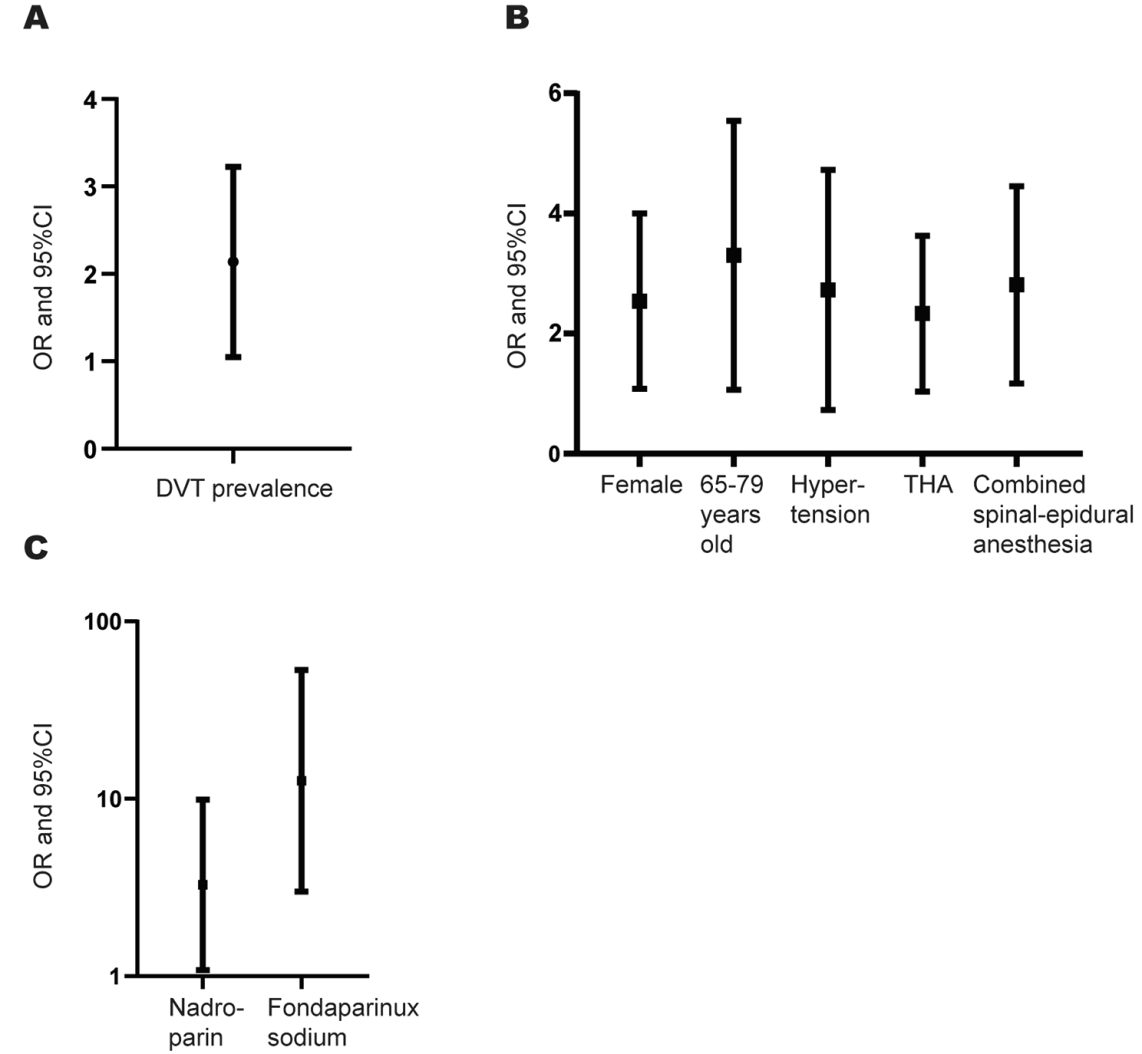
**A**. Nadroparin showed a lower prevalence of DVT than fondaparinux sodium. **B**. Subgroup Analysis of DVT prevalence. **C**. Prevalence of DVT after THA and TKA among patients using the same anticoagulants. Abbreviation: CI, confidence interval; DVT, deep vein thrombosis; THA, total hip arthroplasty ; TKA, total knee arthroplasty

**Fig. 2:**
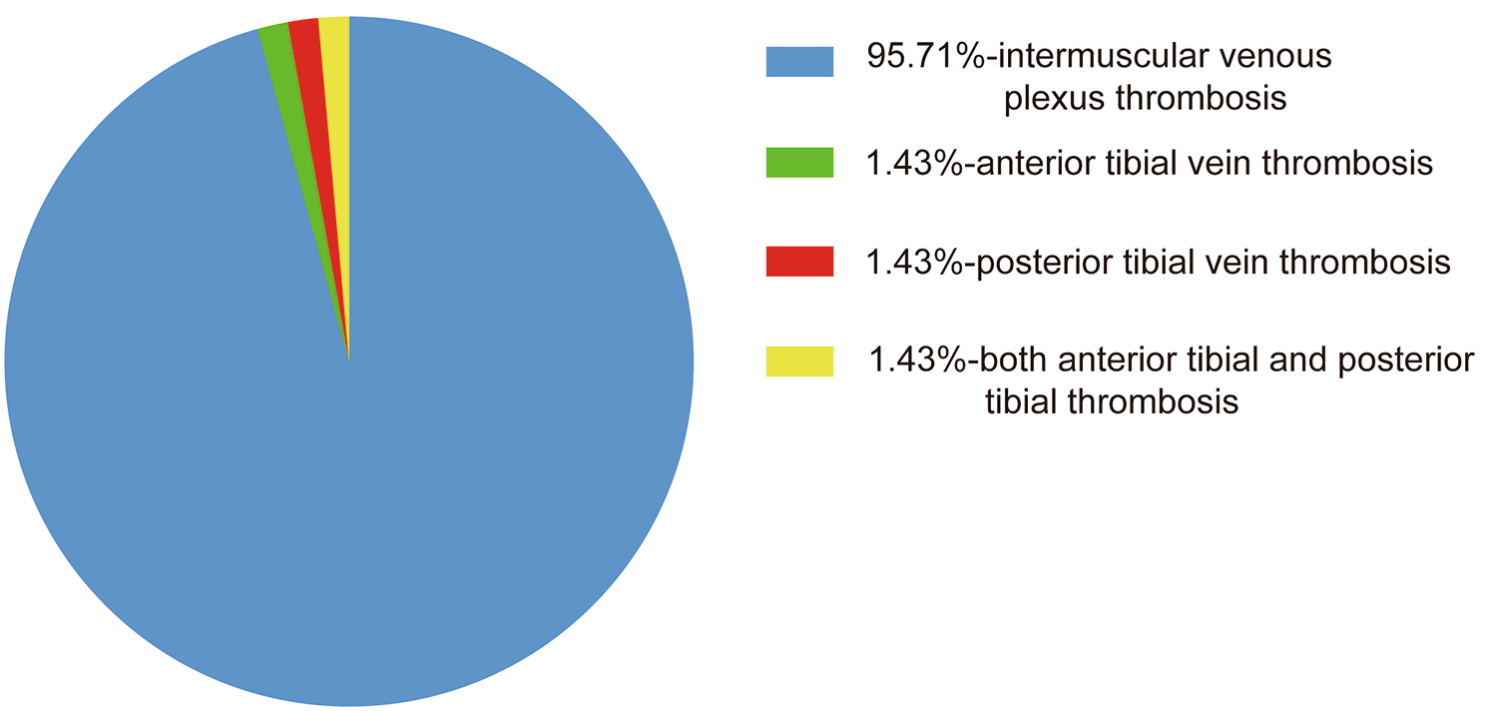
The proportion of the 4 types of thrombosis

A total of 592 patients were included in the study. Among all patients, 70 patients had thrombosis and 522 patients had no thrombosis, and the prevalence of thrombosis was 11.8%. In patients treated with nadroparin, 23 patients experienced thrombosis and 255 patients did not, resulting in a thrombosis rate of 8.3%. Among patients receiving fondaparinux sodium, 47 patients developed thrombosis and 267 patients did not develop thrombosis, with a thrombosis prevalence of 15.0%. Comparing the two anticoagulants, nadroparin showed a lower prevalence of DVT than fondaparinux sodium (8.3% vs. 15.0%, OR = 1.952, 95% CI = 1.152–3.307, *P* = 0.012). The DVT prevalence is illustrated in Table 2 and its OR and 95%CI is illustrated in Fig. 1. Among all patients with DVT, 67 patients developed intermuscular venous plexus thrombosis, 1 patient developed anterior tibial vein thrombosis, 1 patient developed posterior tibial vein thrombosis, and 1 patient developed both anterior tibial and posterior tibial thrombosis. The proportion of the 4 types of thrombosis is illustrated in Fig. 2.

**Table 2:** DVT prevalence

	Nadroparin (*n* = 278) no. (%)	Fondaparinux Sodium (*n* = 314) no. (%)	*P*-value	OR (95% CI)
DVT	23/278(8.3%)	47/314(15.0)	0.012	1.952 (1.152–3.307)

### Subgroup analysis

Statistical analysis of subgroups showed that nadroparin showed a lower prevalence of DVT than fondaparinux sodium in some special patients groups such as female patients (OR = 2.258, 95%CI = 1.238–4.119, *P* = 0.007), patients who are 65–79 years old (OR = 2.796, 95%CI = 1.360–5.751, *P* = 0.004), patients with hypertension (OR = 2.237, 95%CI = 1.016–4.925, *P* = 0.042), patients who underwent TKA (OR = 2.091, 95%CI = 1.172–3.732, *P* = 0.011), and patients who underwent combined spinal-epidural anesthesia (OR = 2.490, 95%CI = 1.352–4.587, *P* = 0.003). In other patients groups such as male patients, patients with diabetes mellitus, patients with coronary heart disease, patients who underwent THA, patients who underwent general anesthesia, there was no statistical significance between two drugs in the prevention of VTE. All statistics is illustrated in Table 3; Fig. 1.

**Table 3:** Subgroup Analysis of DVT in hospital

Outcome	Nadroparin (*n* = 278) no. (%)	Fondaparinux Sodium (*n* = 314) no. (%)	*P*-value	OR (95%CI)
Gender				
Female-no.(%)	17/175(9.7)	43/220(19.5)	0.007	2.258(1.238–4.119)
Male-no.(%)	6/103(5.8)	4/94(4.3)	0.860	-
Age stratification				
≥ 80 year.-no. (%)	4/35(11.4)	5/25(20.0)	0.582	-
65–79 year.-no. (%)	11/160(6.9)	32/187(17.1)	0.004	2.796(1.360–5.751)
<65 year.-no. (%)	8/83(9.6)	10/102(9.8)	0.970	-
Comorbidity				
Hypertension - no. (%)	10/131(7.6)	22/141(15.6)	0.042	2.237(1.016–4.925)
Type 2 diabetes - no. (%)	2/45(4.4)	7/45(15.6)	0.16	-
Coronary heart disease - no. (%)	1/9(11.1)	0/2(0)	-	-
Type of surgery				
THA- no. (%)	1/108(0.9)	2/98(2.0)	0.932	-
TKA- no. (%)	19/170(11.2)	45/216(20.8)	0.011	2.091(1.172–3.732)
Type of anesthesia				
General anesthesia- no. (%)	6/96(6.3)	8/123(6.5)	0.939	-
Combined spinal-epidural anesthesia- no. (%)	17/182(9.3)	39/191(20.4)	0.003	2.490(1.352–4.587)

### VTE prevalence in different type of surgery using the same anticoagulants

Moreover, we also compared whether there was any difference in the prevalence of DVT after THA and TKA among patients using the same anticoagulants. The results showed that among patients using nadroparin, 3.7% of THA and 11.2% of TKA patients developed DVT. Meanwhile, among patients using fondaparinux, DVT occurred in 2.0% of THA patients and 20.8% of TKA patients. Statistical analysis showed there was statistical significance between the prevalence of DVT of two types of surgery, no matter using which anticoagulants. The statistics is illustrated in Table 4; Fig. 1.

**Table 4:** VTE prevalence in different type of surgery using the same anticoagulants

	Type of surgery	DVT	*P*-value	OR (95% CI)
**Nadroparin**	THA- no. (%)	4(3.7)	0.027	3.272 (1.082–9.895)
	TKA- no. (%)	19(11.2)		
**Fondaparinux sodium**	THA- no. (%)	2(2.0)	<0.001	12.632 (2.998–53.221)
	TKA- no. (%)	45(20.8)		

### The independent risk factors of DVT in each group

We also analyzed independent risk factors for DVT in both groups, and logistic regression was used to analyze independent risk factors for DVT within each group. In Table 4 it has been identified that the type of surgery is an independent risk factor for both groups. The independent risk factors in the fondaparinux sodium group are shown in Table 5. In conclusion, the prevalence of DVT was lower in female and in patients underwent combined spinal and epidural anesthesia in the fondaparinux group but not in the nadroparin group. In both groups, patients who underwent THA showed a lower prevalence of DVT.

**Table 5:** Independent risk factors of DVT in fondaparinux sodium group

	*P*-value	OR (95%CI)
Gender (Female*)	0.021	3.667(1.214–11.080)
Type of anesthesia (General anesthesia*)	0.031	0.397(0.171–0.921)

*control group

## Discussion

DVT is common after major surgery or severe trauma, long-term bedridden, limb immobilization and so on [17]. Previous study suggests that the prevalence of DVT after orthopedic surgery varies from 42 to 57% after THA and from 41 to 85% after TKA without thromboprophylaxis [5]. In our study, the prevalence of thrombosis was 11.8% with thromboprophylaxis. Although no study has been reported on nadroparin and fondaparinux sodium for the prevention of lower extremity DVT after orthopedic joint replacement surgery, previously. Nevertheless, our results show that the administration of 4100AxaIU nadroparin significantly reduced the prevalence of postoperative DVT compared with the administration of 2.5 mg fondaparinux in patients undergoing THA and TKA.

A number of randomized controlled clinical trials have shown fondaparinux sodium to be superior to enoxaparin in the prevention of VTE following hip fracture, hip replacement, and major knee surgery [12, 13, 18]. A real-world study conducted in China retrospectively analyzed the prevention of VTE in patients undergoing major orthopedic surgery and trauma, suggesting that fondaparinux sodium may show better thromboprophylaxis effect in hospitalized patients undergoing major orthopedic surgery or trauma than LMWHs, especially in some special patient populations such as elderly patients, patients with renal impairment, patients with hypertension, etc [14]. However, the LMWHs used in this study did not limit the types of anticoagulants (nadroparin or fondaparinux sodium), which may affect the accuracy of the results. Currently, there still lack studies that systematically review the efficacy and safety of enoxaparin and nadroparin in the prevention of VTE after major orthopedic surgery.

The American College of Chest Physicians (ACCP) guidelines recommend the use of LMWH, low-dose unfractionated heparin (UFH), vitamin K antagonist (VKA), fondaparinux, apixaban, dabigatran, rivaroxaban, or aspirin for patients who undergo TKA or THA to prevent VTE [19]. LMWHs are derived from UFH by chemical or enzymatic depolymerization. Enoxaparin is derived from UFH by benzylation followed by alkaline depolymerization, has an average molecular weight of 3500-5500Da, and contains 10,000 International Unit (IU) of anti-Xa factor (AxaIU, WHO compendial units) per ml [11]. Nadroparin is derived from UFH by nitrous acid depolymerization, has an average molecular weight of 3600-5000Da, and contains 10,250 AxaIU per ml [11]. LMWHs exert their primary anticoagulant effect by catalyzing AT-mediated inhibition of coagulation factors. The thromboprophylaxis efficacy of LMWHs is equivalent to that of low-dose subcutaneous UFH, with a lower risk of bleeding complications and fewer heparin-related side effects [20]. Fondaparinux sodium is an artificially isolated natural high-affinity pentasaccharide, synthesized after modification, with a molecular weight of 1728Da [11]. UFH and LMWHs bind to antithrombin III (AT III) and enhance its affinity for thrombin and factor Xa, whereas fondaparinux only enhances the affinity of AT III and factor Xa [11]. Although fondaparinux sodium has been reported to have higher specific anti-Xa activity than LMWHs (approximately 700 units/mg and 100 units/mg, respectively) and a longer half-life after subcutaneous injection than LMWHs (approximately 17 h and 4 h, respectively), Garcia et al. considered that the validity of low molecular weight heparin as a reference preparation to determine fondaparinux sodium anti-Xa activity is doubtful [11].

The ACCP recommends LMWHs as optimal pharmacological agents for VTE prophylaxis in patients undergoing THA, TKA, or hip fracture surgery [21]. According to Yngve et al., based on moderate-quality evidence, the use of fondaparinux sodium compared with LMWHs does not appear to reduce patient-important VTE events but may increase major bleeding events by nine per 1,000 [21]. Besides, The ACCP advised to use fondaparinux sodium cautiously in patients weighing less than 50 kg and elderly and frail patients because bleeding complications may be increased [21].

Subgroup analyses identified a lower prevalence of DVT in the nadroparin group than that in the fondaparinux sodium group in some special patients groups, including female patients, patients who are 65–79 years old, patients with hypertension, patients who underwent TKA, and patients who underwent combined spinal-epidural anesthesia. This may provide an idea for individualized and precise prevention of DVT after THA and TKA. Our results showed that TKA was associated with a higher prevalence of DVT than THA, which is consistent with the results of Senay et al [22]. In their study, the DVT prevalence rates was 2.2% after TKA and 0.4% after THA during hospitalization. Bedsides, a meta-analysis of 47 studies performed by Januel et al. reported that the pooled prevalence rates of VTE for patients treated with LMWHs were 1.4% (95% CI: 1.0–1.8%) in TKA studies and 0.6% (95% CI: 0.4–0.8%) in THA studies [23]. The above studies collected symptomatic VTE, whereas we routinely screened and included many asymptomatic patients with thrombosis, so the prevalence of VTE in our study was higher than the above studies. At the same time, our study found that the prevalence of lower limb intermuscular venous plexus thrombosis was very high, and most of them were asymptomatic. Therefore, attention should be paid to the screening and prevention of lower limb intermuscular venous plexus thrombosis. In an article in the field of cardiovascular disease, the researchers found that direct oral anticoagulants were associated with a lower risk of intracranial hemorrhage and all-cause mortality in female compared with well-controlled warfarin users, but the mechanism was unclear [24]. In our research, its internal mechanism needs to be studied more deeply in the future.

### The limitations of this study

Our study has several limitations and shortcomings. Firstly, it was a single-center retrospective study and selection bias was unavoidable. Secondly, the sample size included in the study was limited, but it is quite enough for the study. Thirdly, we did not use venography but vascular B-ultrasound as the diagnostic standard. Although venography is the gold standard, it is an invasive examination and is not suitable as a screening method. Lower limb vascular B-ultrasound causes little harm to patients and is cheaper. Fourthly, lack of reporting of pulmonary thromboembolic events was also a study limitation, although there were no cases of pulmonary thromboembolism in our study. Finally, there is a lack of comparison of the efficacy of different types of LMWHs.

## Conclusion

In conclusion, the use of 4100AxaIU nadroparin may demonstrate better prevention of DVT in the lower extremities after THA and TKA than 2.5 mg fondaparinux sodium, especially in certain populations such as female patients, patients aged 65–79 years, patients with hypertension, patients underwent TKA, and patients underwent combined spinal-epidural anesthesia. We call for multicenter, large-sample clinical investigations or randomized controlled trials to further elucidate the preventive effects of different anticoagulants on DVT in the lower extremities.

## Data Availability

The data that support the findings of this study are not openly available due to reasons of sensitivity and are available from the corresponding author upon reasonable request.
